# Safety and Feasibility Using a Fluid-Filled Wire to Avoid Hydrostatic Errors in Physiological Intracoronary Measurements

**DOI:** 10.1155/2024/6664482

**Published:** 2024-01-02

**Authors:** Truls Råmunddal, Christian Dworeck, Petronella Torild, Sofie Andréen, Li-Ming Gan, Geir Hirlekar, Dan Ioanes, Anna Myredal, Jacob Odenstedt, Petur Petursson, Tetiana Pylova, Fanny Töpel, Sebastian Völz, Mats Hilmersson, Björn Redfors, Oskar Angerås

**Affiliations:** ^1^Department of Cardiology, Sahlgrenska University Hospital, Gothenburg, Sweden; ^2^Department of Molecular and Clinical Medicine, Institute of Medicine, Gothenburg University, Gothenburg, Sweden; ^3^Cavis Technologies AB, Uppsala, Sweden

## Abstract

**Background:**

Using a fluid-filled wire with a pressure sensor outside the patient compared to a conventional pressure wire may avoid the systematic error introduced by the hydrostatic pressure within the coronary circulation.

**Aims:**

To assess the safety and effectiveness of the novel fluid-filled wire, Wirecath (Cavis Technologies, Uppsala, Sweden), as well as its ability to avoid the hydrostatic pressure error.

**Methods and Results:**

The Wirecath pressure wire was used in 45 eligible patients who underwent invasive coronary angiography and had a clinical indication for invasive coronary pressure measurement at Sahlgrenska University Hospital, Gothenburg, Sweden. In 29 patients, a simultaneous measurement was performed with a conventional coronary pressure wire (PressureWire X, Abbott Medical, Plymouth, MN, USA), and in 19 patients, the vertical height difference between the tip of the guide catheter and the wire measure point was measured in a 90-degree lateral angiographic projection. No adverse events caused by the pressure wires were reported. The mean Pd/Pa and mean FFR using the fluid-filled wire and the sensor-tipped wire differed significantly; however, after correcting for the hydrostatic effect, the sensor-tipped wire pressure correlated well with the fluid-filled wire pressure (*R* = 0.74 vs. *R* = 0.89 at rest and *R* = 0.89 vs. *R* = 0.98 at hyperemia).

**Conclusion:**

Hydrostatic errors in physiologic measurements can be avoided by using the fluid-filled Wirecath wire, which was safe to use in the present study. This trial is registered with NCT04776577 and NCT04802681.

## 1. Introduction

Using intracoronary pressure measurements to evaluate the clinical significance of coronary stenosis is recommended in European and American guidelines [1, 2]. The most common method to perform a pressure measurement is fractional flow reserve (FFR) using adenosine to accomplish hyperemia; however, resting indices, e.g., instant wave-free ratio (iFR), have been proven as effective as FFR to distinguish a significant from a nonsignificant lesion [3].

**Figure 1:**
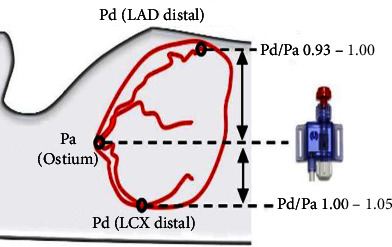
Hydrostatic effects in ∼99% of the population. Illustration of the influence of the height difference between the coronary ostia and distal coronary arteries and the measured pressure.

Several coronary wires are available to measure pressure inside the coronary artery, all with a pressure sensor in the distal part of the wire. The pressure sensor is calibrated to the aortic pressure by equalization of pressures when the pressure is measured at the same position, i.e., outside the guide catheter in the proximal part of the vessel or in the aorta. However, when advancing the wire into the coronary vessel, the hydrostatic pressure, due to the height difference between the coronary ostium and the distal part of the vessel, causes an unaccounted influence on the measured pressure. In a supine position, the left anterior descending artery (LAD) and posterior descending artery from the right coronary artery (PDA) ascend on average 5 cm, and the left circumflex artery (LCX) and posterior lateral artery from the right coronary artery (PLA) descend on average 3 cm (Figure 1). This results in a decrease in the measured pressure in the LAD and the PDA and an increase in pressure in LCX and PLA due to the hydrostatic pressure. CT studies measuring this height difference suggest a conversion factor of 0.77 mmHg/cm in order to adjust for differences in hydrostatic pressure [4–6].

The systematic error in conventional pressure measurements introduced by the hydrostatic pressure has been described previously but has not been accounted for in studies or guidelines when suggesting clinically used pressure ratio cutoffs for significance pre- and post-PCI regardless of type of pressure index [4].

**Figure 2:**
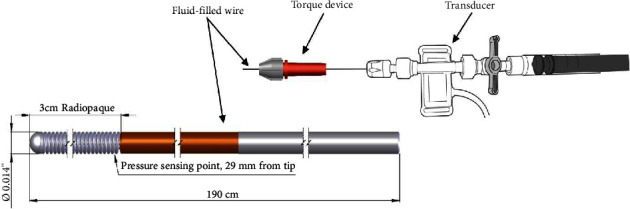
Wirecath pressure wire with external transducer. Illustration of the design of the fluid-filled wire and transducer.

The Wirecath (Cavis Technologies, Uppsala, Sweden) (Figure 2) is a novel fluid-filled 0.014″ wire connected to an external pressure transducer with fixed height which makes the measurements by the Wirecath unaffected by the hydrostatic pressure difference in the coronary artery [7].

This first-in-human Wirecath study (ClinicalTrials.gov Identifier: NCT04776577) was designed to assess the safety and effectiveness of the novel fluid-filled wire as well as its ability to avoid the hydrostatic pressure error. The study was approved by the Swedish Ethical Review Authority (D-nr 2020-04421).

## 2. Methods

### 2.1. Patient Selection

Patients were recruited at Sahlgrenska University Hospital, Gothenburg, Sweden. Inclusion criteria were age 18 or above, signed informed consent, and clinical indication for coronary pressure assessment after coronary angiography. Exclusion criteria were known heparin-induced thrombocytopenia, other allergy to heparin, contraindication for adenosine, or unstable condition by operator´s judgment.

### 2.2. Study Design

**Figure 3:**
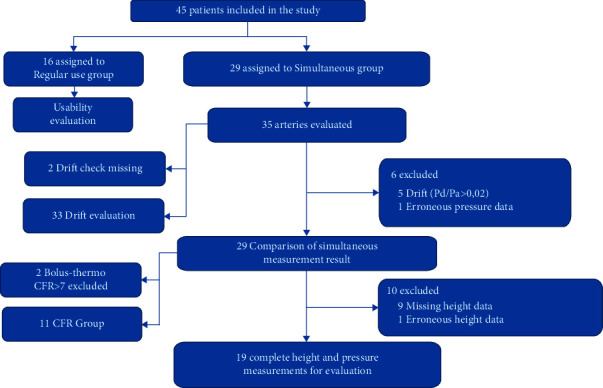
Enrollment flowchart.

As outlined in Figure3, 29 patients underwent simultaneous measurement with the fluid-filled wire (Wirecath 1, Cavis Technologies, Uppsala, Sweden) and a sensor-tipped wire (PressureWire X, Abbott Medical, Plymouth, MN, USA). At the discretion of the physician, 11 out of these 29 patients (38%) underwent bolus-thermo-derived and within 14 days transthoracic ultrasound Doppler-derived coronary flow reserve (CFR) measurements in the LAD.

An additional 16 patients were enrolled who had coronary physiology assessment only using the fluid-filled wire (Wirecath 1, Cavis Technologies, Uppsala, Sweden). After each of the 45 procedures, a usability questionnaire pertaining to the use of the fluid-filled wire was filled out by the users.

The primary endpoint was the rate of adverse events, defined as an untoward medical occurrence, unintended disease or injury, or untoward clinical sign in any of the 45 studied subjects. The key secondary endpoints were the agreement between Pd/Pa and FFR measured with the fluid-filled (Wirecath) and sensor-tipped (Abbott PressureWire) before and after correction for the hydrostatic pressure by using the height measurements. Exploratory secondary endpoints included the correlations between fluid-filled and sensor-tipped wire-derived indices with CFR indices and the usability questionnaire score.

### 2.3. Procedure

Angiography was performed according to the local standard procedure. All patients received nitroglycerine prior to all physiology assessment. Hyperemia was induced by administering adenosine intravenous (140 *μ*g/kg/min over two minutes) or intracoronary (200 *μ*g for the left coronary vessel and 120–200 *μ*g for the right coronary vessel). Whether the patients had simultaneous measurements with the fluid-filled and sensor-tipped wires or only had coronary physiology assessment using the fluid-filled wire was left to the discretion of the physician performing the procedure.

### 2.4. Simultaneous Measurements

The fluid-filled wire (Wirecath 1, Cavis Technologies, Uppsala, Sweden) and the sensor-tipped wire (PressureWire X, Abbott Medical, Plymouth, MN, USA) were placed simultaneously at the same location in the coronary artery. Simultaneous pressure measurements were then performed with both wires, both resting Pd/Pa and FFR.

**Figure 4:**
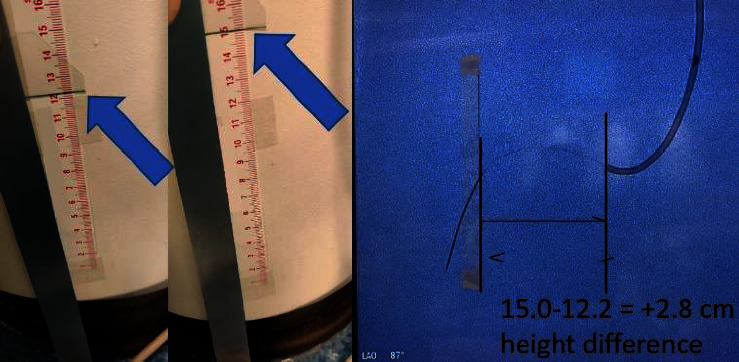
Height measurement between the pressure measure point and the tip of the guide catheter. Example of an observed height difference between the pressure measure point and the tip of the guide catheter.

The vertical height difference between the tip of the guide catheter and the wire measure point was measured in a 90-degree lateral angiographic projection by first marking the position of the guide catheter tip with adhesive tape on the fluoroscopy screen, then adjusting the vertical position of the x-ray table until the measuring point of the wire on fluoroscopy reaches the tape mark on the screen, and finally assessing the total adjusted distance in centimeter between the two table positions on a tape measure attached to the patient table (Figure 4).

When the wires were withdrawn from the artery into the tip of the guide catheter, the observed “Pd/Pa” was recorded to allow quantification of any drift in pressure.

By multiplying each height difference, negative or positive, with the conversion factor 0.77 mmHg/cm, a correction for each pressure measurement by the sensor-tipped wire was established. By adjusting the sensor-tipped pressure with this correction, the pressure from the sensor-tipped wire was corrected for the hydrostatic effect.

### 2.5. Coronary Flow Reserve

A subset of patients who had simultaneous measurements by the fluid-filled and pressure-tipped wires underwent bolus-thermo-derived coronary flow reserve (CFR) measurements in the LAD and was subsequently referred to transthoracic Doppler-echocardiography-derived CFR measurement (TDE-CFR), which has shown to be highly correlated with CFR derived from positron emission tomography [8] and to invasive Doppler guide wire-derived CFR [9, 10]. TDE-CFR was performed according to the standard procedure of the hospital [11]. The correlation between a pressure-derived CFR, bolus-thermo-derived CFR, and the echocardiography-CFR was investigated. To mimic the situation during the invasive physiology measurement, all patients received the same nitroglycerin dose 5 min prior to the TDE-CFR assessment measured during adenosine hyperemia (140 *μ*g/kg/min). Pressure-derived CFR was calculated by the formula 1-FFR/1-Pd/Pa [12].

### 2.6. Regular Use

In the regular-use study group, the fluid-filled wire was used as a regular pressure measurement tool, replacing the standard pressure wire.

### 2.7. Statistical Methods

The results are presented with descriptive statistics. Unless otherwise noted, all summed values are presented as mean ± standard deviation. In the comparison of pressure measurements, the Pearson correlation factor and the intraclass correlation coefficient were calculated and regression plots were made for paired measurements. The CFR measurement comparisons were exploratory only and intended to give a first insight into the capability of pressure-derived CFR with a fluid-filled wire.

## 3. Results

Patients' age ranged from 54 to 82 years (mean 70 ± 6.5 years), and 31% were female. The indications for angiography were 14 patients with stable angina, 9 with unstable angina, 7 with non-ST elevation myocardial infarction, and the remaining with other indications.

No adverse events related to the use of the pressure wires were reported.

### 3.1. Comparisons of Pd/Pa and FFR after Simultaneous Measurements with Fluid-Filled and Sensor-Tipped Wires

35 coronary arteries in 29 patients were included in the simultaneous measurements study group only.

24 lesions (69%) were located in the LAD artery, 4 (11%) in the LCX, and 7 (20%) in the right coronary artery (RCA).

The mean Pd/Pa at rest in the 29 simultaneous measurements, after removal of five drift cases (Pd/Pa >0.02) and one erroneous pressure data, using the fluid-filled wire and the sensor-tipped wire were 0.96 ± 0.03 and 0.94 ± 0.04, respectively. The mean FFR in the same 29 simultaneous measurements using the fluid-filled wire and the sensor-tipped wire were 0.89 ± 0.07 and 0.87 ± 0.07, respectively.

Successful height measurements were collected in 19 simultaneous measurements, after the exclusion of nine cases with missing height data and one erroneous height measurement. Data were missing due to difficulties in visualizing both the guide catheter tip and the wire position in the lateral view.

The 19 arteries consisted of 14 LAD, 2 LCX, and 3 RCA. The mean difference in height between the coronary ostium and the pressure measure point was 2.6 cm, corresponding to a mean hydrostatic pressure difference of 2.0 mmHg, using the conversion factor 0.77 mmHg/cm [6].

Table 1 summarizes the results showing that the difference between the two wires diminished when the sensor-tipped wire measurements were corrected.  Table 2 presents which coronary vessel was measured, measured height difference, calculated hydrostatic pressure, and aortic and coronary pressure ratios.

**Figure 5:**
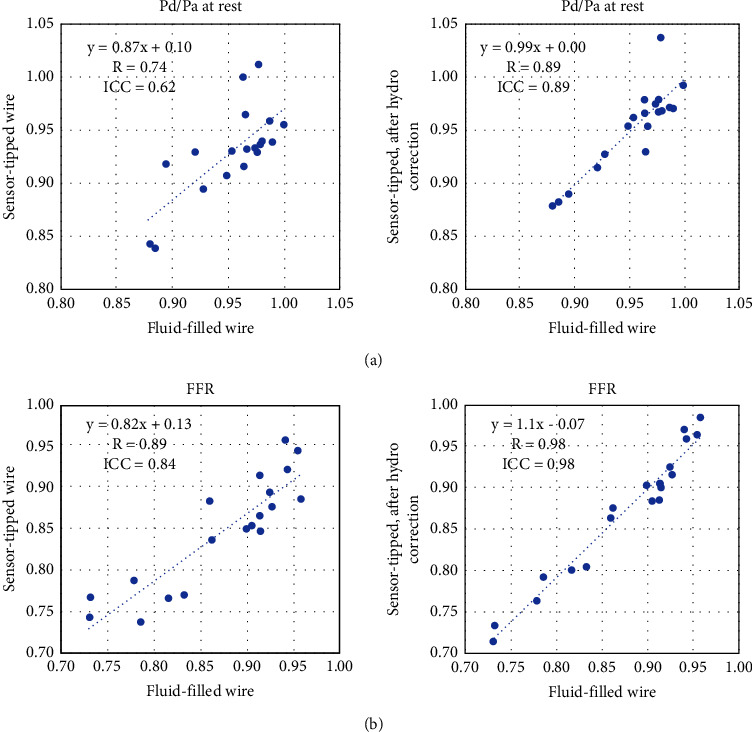
Correlation between indices derived using the fluid-filled versus sensor-tipped wires. The correlation for both Pd/Pa (a) and fractional flow reserve (FFR) (b) between the two wires was greater when the hydrostatic error due to height was accounted for (right versus left). Passing-Bablok regression indicates that a small proportional bias was present between FFR derived using the fluid-filled wire versus the sensor-tipped wire after correction for hydrostatic pressure (slope 1.14 (95% CI) [1.01–1.38]) but not in the other comparisons.

Figure 5 shows that after correcting for the hydrostatic effect, the sensor-tipped wire pressure correlated well with the fluid-filled wire pressure (*R* = 0.74 vs. *R* = 0.89 at rest and *R* = 0.89 vs. *R* = 0.98 at hyperemia).

In the subgroup where measurements were made in the LAD, the mean Pd/Pa at rest were 0.96 ± 0.04 and 0.92 ± 0.04 for the fluid-filled wire and the sensor-tipped wire, respectively, and the mean FFR were 0.90 ± 0.05 and 0.86 ± 0.06, respectively.

Out of the 29 valid measurements, the number of discordant classifications between the sensor-tipped wire and the fluid-filled wire was explored for the Pd/Pa at rest and the FFR measurements.

When applying the cutoff Pd/Pa ≤ 0.92 [13], two LADs were significant according to the sensor-tipped wire but not according to the fluid-filled wire. Two RCAs were significant according to the fluid-filled wire but not according to the sensor-tipped wire.

When applying the cutoff FFR ≤ 0.80, three LADs were significant according to the sensor-tipped wire, but not according to the fluid-filled wire.

Drift was compared in 33 simultaneous measurements in 27 patients. The fluid-filled wire demonstrated less drift than the sensor-tipped wire (standard deviation 0.11 vs. 0.18). With an increasing number of cases, less drift was observed, possibly learning curve-related.

### 3.2. Correlations between Pressure Indices and CFR

**Figure 6:**
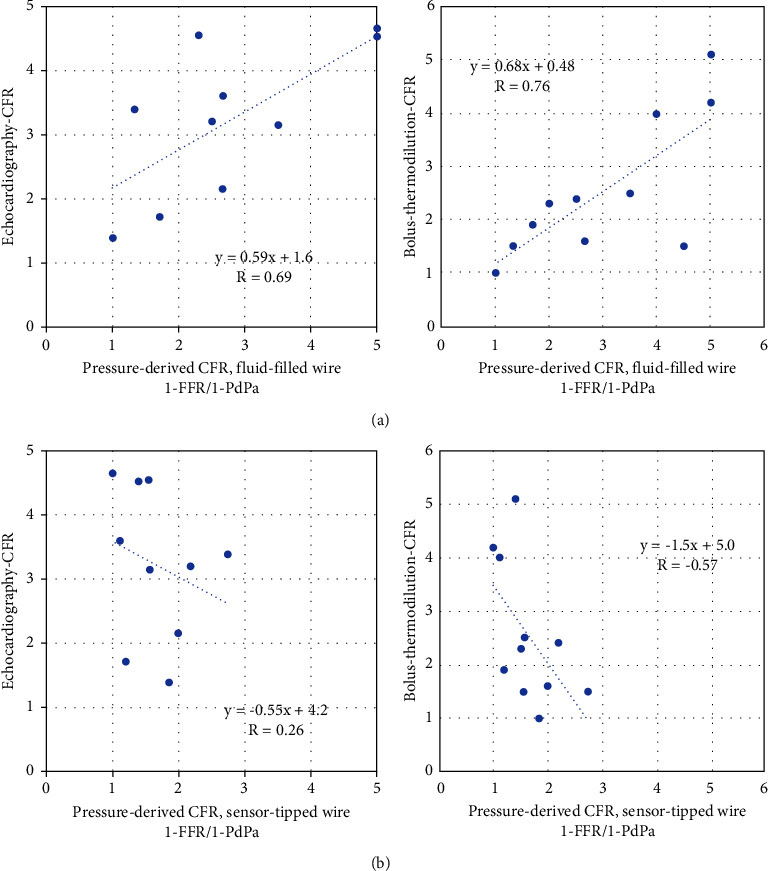
Correlation between pressure-derived coronary flow reserve (CFR) and CFR derived by echocardiography or thermodilution. (a) Correlation between pressure-derived CFR using the fluid-filled wire and CFR derived by echocardiography (left) and thermodilution (right). (b) Correlation between pressure-derived CFR using the sensor-tipped wire and CFR derived by echocardiography (left) and thermodilution (right).

Pressure-derived CFR with the fluid-filled wire correlated to TDE-CFR and thermodilution-CFR (*R* = 0.69 and *R* = 0.76, respectively (Figure 6(a))). Sensor-tipped wire pressure-derived CFR did not correlate positively to the reference methods; see Figure 6(b). When using the square root of 1-FFR/1-PdPa, relationships get less proportional, and R values do not improve compared to not using square root.

### 3.3. Results of the Usability Questionnaire

16 patients were included in the regular-use study group only. The fluid-filled wire was used as a regular pressure measurement tool, and in two cases, the fluid-filled wire was used for balloon and stent intervention during PCI.

Usability was evaluated by using a questionnaire filled in by the interventionalists in this group. The scoring was similar for the fluid-filled wire and the sensor-tipped wire when it came to perceived maneuverability, X-ray visibility, and signal quality. The preparation of the fluid-filled wire had a mean score of 2.8 compared to the sensor-tipped wire 3.0, on a scale from 1 (poor) to 5 (very good).

## 4. Discussion

The main findings of this study are that (i) the fluid-filled Wirecath wire (Cavis Technologies, Uppsala, Sweden) could be safely and reliably used to assess Pd/Pa and FFR for clinical decision-making in a typical patient population undergoing PCI; (ii) the differences between FFR and resting indices obtained by the sensor-tipped versus fluid-filled wires were explained by a hydrostatic pressure component related to the height difference between the coronary ostium and the distal vessel; and (iii) correcting the sensor-tipped values for the height difference almost completely aligned measurements derived by the sensor-tipped versus fluid-filled wires.

The hydrostatic pressure difference (which is the same value for resting and hyperemia measurements) has a larger impact on resting ratios since the true pressure reduction across the lesion is smaller at rest than during hyperemia (FFR). The effect is also larger on diastolic resting ratios compared to whole-cycle ratios, since the fixed hydrostatic pressure difference is relatively larger in diastolic versus mean blood ratios [5, 14].

A neglection of the hydrostatic error may have implications for clinical practice. The hydrostatic error causes lower measured values (i.e., overestimation) in the LAD or distal PDA, since these vessels in a supine patient position are located higher than the coronary ostium, i.e., the hydrostatic pressure falls. This measurement error might lead to false-positive pressure ratios and thus misclassification as clinically significant. Vice versa may the rise in hydrostatic pressure cause an underestimation in non-LAD vessels. This could be of special importance in patients where FFR is used to evaluate the result of the PCI exposing the patient to the risk of leaving an inadequate result or the risk of complication when trying to improve an already adequate stenting [15].

Coronary CT has previously been used for the measurement of height differences in coronary vessels [4, 5]. Another potential way to calculate the hydrostatic error, when a fluid-filled wire is not available, is to use quantitative coronary angiography (QCA) tools to measure the height in a lateral angiographic view and compensate for the height difference between the tip of the guide catheter and the measure point of the wire. This would simplify the height measurement compared to the method used in this study. The QCA method is currently evaluated in another study (ClinicalTrials.gov Identifier: NCT04802681).

When using a fluid-filled wire, pressure-derived CFR correlates to other CFR measurement methods as opposed to when using a sensor-tipped wire. This is presumably due to the avoidance of the hydrostatic error, which has also been shown in other explorative studies [16]. Due to the small sample size, no firm conclusion can be drawn, but the result may suggest that pressure-derived CFR can possibly be used as a screening tool for evaluating CFR and other CFR-related indices such as resistive reserve ratio (RRR) and microvascular resistance reserve (MRR).

### 4.1. Limitations

A larger dataset than available in this study would be needed to statistically evaluate the amount of discordance and misclassification caused by hydrostatic errors. Quantification of the size of errors caused by hydrostatic pressure has been explored in other studies [4, 5].

The reasons for drift were not explored in this study. Drift could be caused by both technical shortcomings of the measurement devices, but also by user mistakes caused by pitfalls when doing pressure measurements [7].

## 5. Conclusions

Hydrostatic pressure introduces error in conventional intracoronary pressure measurements that has a particularly large impact on resting indices. Hydrostatic errors in physiologic measurements can be avoided by using the fluid-filled Wirecath wire, which was safe to use in the present study.

## Figures and Tables

**Table 1:** Summary of simultaneous measurements including height measurements (*n* = 19).

Index		Sensor-tipped wire	Fluid-filled wire	Difference between sensor-tipped and fluid-filled wire	Difference between sensor-tipped and fluid-filled wire after correction of the sensor-tipped wire for the hydrostatic effect
Pd/Pa	Mean ± SD	0.93 ± 0.041	0.95 ± 0.035	0.024 ± 0.028	0.002 ± 0.018
	Median	0.93	0.97	0.036	0.003
	Min	0.84	0.88	−0.04	−0.06
	Max	1.01	1.00	0.05	0.03

FFR	Mean ± SD	0.85 ± 0.066	0.87 ± 0.072	0.026 ± 0.033	0.0037 ± 0.017
	Median	0.85	0.91	0.032	0.0016
	Min	0.74	0.73	−0.035	−0.027
	Max	0.96	0.96	0.072	0.03

**Table 2:** Height measurements and correction of sensor-tipped wire measurements (*n* = 19).

Vessel	Height difference ± *x* (cm)	Hydrostatic error (0.77 mmHg/cm) (mmHg)	Pa at rest sensor-tipped (mmHg)	Pd at rest sensor-tipped (mmHg)	Pd at rest sensor-tipped after hydro correction (mmHg)	Pa at hyperemia sensor-tipped (mmHg)	Pd at hyperemia sensor-tipped (mmHg)	Pd at hyperemia sensor-tipped after hydro correction (mmHg)
LAD	−12.4	−9.5	95	89	98.5	97	86	95.5
LAD	−5.8	−4.5	98	89	93.5	84	62	66.5
LAD	−5.5	−4.2	83	76	80.2	80	68	72.2
LAD	−5.2	−4.0	124	111	115.0	120	92	96.0
LAD	−4.9	−3.8	87	73	76.8	97	85	88.8
LAD	−4.3	−3.3	90	86	89.3	89	82	85.3
LAD	−4.2	−3.2	85	79	82.2	96	74	77.2
LAD	−4.0	−3.1	99	93	96.1	82	71	74.1
LAD	−4.0	−3.1	76	71	74.1	59	50	53.1
LAD	−3.8	−2.9	83	70	72.9	73	61	63.9
LAD	−3.7	−2.8	100	94	96.8	94	84	86.8
LAD	−3.6	−2.8	86	80	82.8	96	82	84.8
LAD	−2.4	−1.8	89	83	84.8	89	84	85.8
LAD	−1.2	−0.9	73	70	70.9	68	65	65.9
RCA	2.1	1.6	113	105	103.4	66	52	50.4
LCX	2.3	1.8	82	82	80.2	86	76	74.2
RCA	3.0	2.3	85	78	75.7	78	58	55.7
RCA	3.7	2.8	86	87	84.2	94	86	83.2
LCX	3.9	3.0	86	83	80.0	86	66	63.0

## Data Availability

The study data used to support the findings of this study are available from the corresponding author upon request.
